# Viral suppression and adherence in adolescents living with HIV in rural Tanzania

**DOI:** 10.1371/journal.pone.0315866

**Published:** 2024-12-20

**Authors:** Ezekiel Luoga, James Okuma, Lilian Moshi, George Sigalla, Dorcas Mnzava, Daniel H. Paris, Tracy R. Glass, Fiona Vanobberghen, Maja Weisser, Getrud Joseph Mollel, on behalf of the KIULARCO working group

**Affiliations:** 1 Ifakara Health Institute, Ifakara, Tanzania; 2 St. Francis Referral Hospital, Ifakara, Tanzania; 3 Swiss Tropical and Public Health Institute, Allschwil, Switzerland; 4 University of Basel, Basel, Switzerland; 5 Division of Infectious Diseases and Hospital Epidemiology, University Hospital Basel, Basel, Switzerland; Ethiopian Public Health Institute, ETHIOPIA

## Abstract

**Background:**

Adolescents living with HIV (ALHIV) in sub-Saharan Africa are affected by poor treatment outcomes, likely a consequence of poor adherence.

**Objectives:**

To assess viral suppression rates and evaluate factors associated with achieving viral suppression and maintaining treatment adherence among ALHIV in rural Tanzania.

**Methods:**

Cross-sectional analysis of data from the Kilombero and Ulanga Antiretroviral Cohort in Ifakara, Tanzania, including adolescents aged 10–19 years on antiretroviral treatment (ART) ≥6 months at the time point of their first viral load (VL) measurement after implementation of routine VL testing from August 2017 through December 2023. VL ≥1000 copies/ml was considered unsuppressed. We assessed agreement between adherence measures (self-report, pill box return, pill count and visual analogy scale) and viral suppression. Logistic regression was used to determine factors associated with viral suppression.

**Results:**

Of 319 included adolescents, 159 (50%) were male, 143 (45%) aged 10–13 years, 213 (74%) had disclosed their HIV status, 72 (23%) lived ≥50 kilometers from the clinic, 161 (55%) had a WHO stage III/IV and 80 (33%) had CD4 cell counts <500 cells/mm^3^. Overall, 249 (78%) adolescents were virally suppressed. Factors associated with viral suppression were having a CD4 cell count ≥500 cells/mm^3^ (adjusted Odds Ratio (aOR) 3.48; 95% CI 1.49–8.13) versus those with a CD4 cell count <500 cells/mm^3^, being on a dolutegravir-based regimen (aOR 12.6; 95% CI 2.50–68.7) versus those on a NNRTI based regimen. Female gender was associated with lower odds of having viral suppression (aOR 0.41; 95%CI 0.18–0.93). There was a weak to moderate agreement between adherence measures and VL suppression.

**Conclusion:**

Adolescents in this rural cohort remain far behind the UNAIDS 95% viral suppression target with only 78% being virally suppressed. The weak to moderate associations between adherence assessment and viral suppression. Adolescents’ HIV care models need to be strengthened in order to achieve viral suppression goals in this population.

## Introduction

Adolescents living with HIV (ALHIV) are disproportionally affected by HIV. In 2023, 1.9 million (1.1–2.5) adolescent girls and young women aged 15–24 years and 1.2 millions (0.8–1.6) boys and young men in the same age category were living with HIV [1]. In Eastern and Southern Africa, numbers of new infections in adolescents remain high, with 27% of the 450,000 new infections in 2023 being among adolescent girls and young women [1]. Tanzania harbours 6% of the global ALHIV population with girls [2].

In programmatic settings such as Tanzania, routine viral load (VL) monitoring was introduced in 2017 [3] with yearly measurements for suppressed people living with HIV (PLHIV) defined as VL below 1000 copies/ml [4, 5]. Viral load suppression rates among ALHIV vary according to settings. A large-scale study from routine care among over 9,000 adolescents aged 10–19 in South Africa reported a viral suppression rate of 74% [6]; studies from Uganda and Kenya have reported viral suppression rates of around 80% [7, 8] and a study from Cameroon reported only 66% suppression in adolescents [9]. Most important factors for poor viral suppression among ALHIV were male gender, rural residence, WHO stage III/IV, CD4 count <500 cells/mm^3^, and poor adherence [6–9]. In addition, ALHIV might be affected by age-related uncertainties and self-stigmatization [10, 11] leading to struggles with antiretroviral treatment (ART) adherence and with poor treatment outcomes [12–14].

Measurement of adherence to ART—crucial for predicting viral suppression, preventing viral resistance, reducing morbidity and mortality, and preventing transmission—remains challenging. Methods such as self-report and pill counts are frequently used due to their ease in implementation but are not very unreliable and subject to recall bias [15–18]. Serum drug concentration determination and electronic adherence monitoring are more objective measurements, but are mostly not available in routine care [19, 20]. Given the variability in ART adherence among adolescents, there is a strong need for a gold standard assessment method to effectively support and improve treatment outcomes [21].

In this study, we determined viral suppression rates, adherence measured by different methods and associated factors among adolescents aged 10–19 years enrolled in the Kilombero and Ulanga Antiretroviral Cohort (KIULARCO) in rural South Western Tanzania, at the time point of their first VL measurement after implementation of routine VL monitoring. Further, we assessed the correlation between adherence measures (self-report, pill box return, pill count and visual analogue scale (VAS)) and viral suppression.

## Methods

### Study design and patient population

This is a cross-sectional analysis of data from KIULARCO, a single-centre, open and ongoing prospective cohort of people living with HIV established in 20005 at the Chronic Diseases Clinic of Ifakara (CDCI) within the Saint Francis Regional Referral Hospital (SFRRH) in Ifakara, Tanzania. From KIULARCO we selected ALHIV aged 10–19 years being on ART for ≥6 months at the timepoint of their first VL measurement after the roll-out of routine VL monitoring with a clinical visit between August 2017 and December 2023. Data was accessed on 31 December 2023. VL conducted on December 31 ware careful reviewed to ensure that all relevant results were included in a dataset and post-extraction data checks was performed to capture VL results that may have been reported after data extraction date.

### Study site

The CDCI is a rural HIV and tuberculosis treatment centre at the SFRRH, which was established in 2005 as a collaboration with the Ifakara Health Institute, the Swiss Tropical and Public Health Institute and the University Hospital Basel, Switzerland. All patients are asked for consent to participate in the KIULARCO, aiming at determining clinical outcomes of HIV care, including opportunistic infections and co-morbidities, monitor adherence, side effects of and toxicity of ART, allowing research based on biobanked plasma samples and provide a platform for studies on improving HIV treatment and care in Sub-Saharan Africa [22, 23]. Patients’ demographics, clinical and laboratory information are captured in an open medical electronic record system (OpenMRS; https://openmrs.org/). The electronic patient database at the CDCI improves healthcare delivery through standardized data assessement and simultaneously is a platform for research activities. Demographic information is entered by data officer, vital signs by nurses, clinical information are entered by clinicians during consultations, and laboratory values are entered by the laboratory staff. Patient management is in accordance to the Tanzania National AIDS Control Program guidelines [24].

### Data source

We extracted clinical and demographic characteristics, and viral load data from the OpenMRS database. We collected additional adherence data including a VAS for a subset of patients, who attended the clinic from February to April 2018 during routine visits. VAS assess adherence of ART by asking patient to give the percentage of adherence on scale ranging from 0 to 100%. Data from VAS were entered in a spreadsheet and merged with OpenMRS database.

### Study variables and outcomes

The primary outcome was the first VL result obtained after implementation of routine VL testing. VL ≥1000 copies/ml was categorised as unsuppressed in accordance with the Tanzania National AIDS Control Guidelines [3, 24].We extracted adherence data for visits matching the VL sample collection date. Self-reported adherence was measured by questioning on any ART doses missed and if yes, on missing more than one dose in a row over the past four weeks. Information about pill box return was extracted for the same drug refill/clinical visit. Pill count among those who returned their pill box was calculated as the percentage of pills taken (dispensed from previous visit minus–returned pills in current visit) divided by number of days elapsed since the last visit. A pill intake of ≥90% was defined as good adherence [25]. A VAS was used in a subset of patients to assess self-rating of adherence, whereby adherence ≥90% was ranked as optimal. VAS was administered for three months only (February to April 2018) with an assumption that most of the adolescents attended their clinic visits three monthly as scheduled.

All covariates were assessed the time of VL testing and included sex, age, years since ART initiation, highest education level, disclosed HIV status, presumed mode of infection, distance in kilometres between residence and the clinic [26, 27], HIV WHO stage and CD4 cell count within 6 months before and up to 3 months after the VL testing, ART regimen [28]. The outcome were viral suppression and adherence.

### Statistical analysis

Demographic and other clinical characteristics were described using numbers and percentages for categorical variables, and medians and interquartile ranges for continuous variables. We compared descriptive characteristics between ALHIV with and without a viral suppression. Logistic regression was used to determine factors associated with viral suppression, reporting odds ratios (OR) and 95% confidence intervals (CI). We fitted univariable and a full multivariable model on complete data of the covariates which were selected a priori. For continuous covariates, we checked for non-linearity using fractional polynomial models. As a sensitivity analysis, we also fitted a multivariable model including covariates with missing values, using missing indicators in order to include all participants in the models (i.e., participants with missing data for a given variable were classified in a separate ‘missing’ category). Associations between different measurements of ART adherence (self-report, pill box return, pill count and VAS) and viral suppression were determined using percent agreement and kappa statistics. All analyses were performed using Stata version 16 (StataCorp LP, College Station, TX, USA).

### Ethical consideration

This study is an analysis of data from KIULARCO, that has been approved by the Ifakara Health Institute- Institutional Review Board (IHI/IRB/No16-2006) and the National Institute of Medical Research (NIMR/HQ/R.8a/Vol.IX/620) with yearly extensions.

## Results

### Patient characteristics

**Fig 1:**
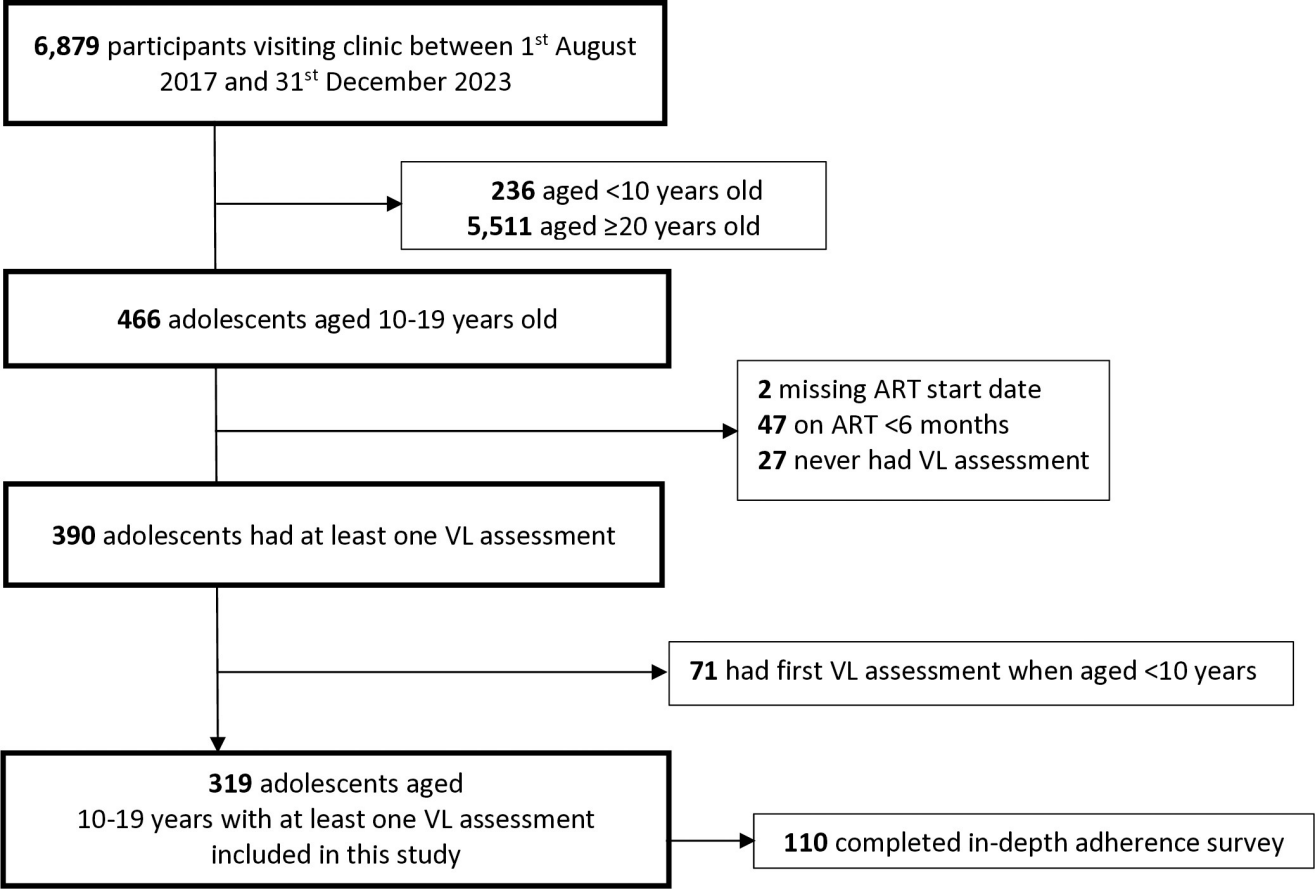
Flow chart of adolescents in KIULARCO cohort.

A total of 466 adolescents aged 10–19 years had clinical visits between August 2017 and December 2023. After exclusion of 147 adolescents (47 were on ART for less than six months, 2 had no ART start date, 27 did not have a VL measurement during the study period and 71 were aged <10 years at the time of the first VL measurement), we included 319 adolescents in the analysis. Of those, 110 (35%) of them completed a VAS assessment of adherence (Fig 1).

The median age was 14 years (interquartile range (IQR) 12–17), 159 (50%) were male, 56 (18%) had no formal education, 213 (74%) had disclosed their HIV status, and 72 (23%) were residing ≥50 kilometres away from the clinic (**Table 1**). The presumed mode of transmission was perinatal in 108 (57%) adolescents. The median time since ART initiation to VL testing was 3.1 years (IQR 0.5–6.9), 161 (55%) adolescents had a WHO stage III/IV, 80 (33%) had a CD4 count <500 cells/mm^3^ and 56 (18%) were on a dolutegravir-based regimen.

**Table 1:** Demographic and clinical characteristics among adolescents living with HIV.

Characteristics	All (N = 319)	Suppressed VL (<1000 copies/ml)	Unsuppressed VL (≥1000 copies/ml)
		N = 249 (78%) N = 70 (22%)	
Sex, n (%) Male			
	159 (50%)	129 (52%)	30 (43%)
Female	160 (50%)	120 (48%)	40 (57%)
Age in years, n (%) 10– 13			
	143 (45%)	112 (45%)	31 (45%)
14–16	87 (27%)	67 (27%)	20 (28%)
17–19	89 (28%)	70 (28%)	19 (27%)
Years since ART initiation, median(IQR)	3.1 (0.5–6.9)	3.0 (0.4–7.2)	3.4 (1.1–6.2)
Education status, n (%) None			
	56 (18%)	46 (19%)	10 (14%)
Primary	225(71%)	171 (71%)	47 (67%)
Secondary	38 (12%)	25 (10%)	13 (19%)
Disclosed HIV status, n (%) No			
	74 (26%)	64 (29%)	10 (16%)
Yes	213 (74%)	159 (71%)	54 (85%)
Missing	32 (10%)	26 (10%)	6 (9%)
Presumed mode of transmission, n (%)			
Perinatal	108 (57%)	79 (54%)	29 (64%)
Heterosexual	47 (25%)	38 (26%)	9 (20%)
Unknown/other Missing	36 (19%)	29 (20%)	7 (18%)
	128 (40%)	103 (41%)	25 (36%)
Distance of residence from clinic, n (%) <1 km			
	137 (44%)	112 (46%)	25 (37%)
2 - <50 km	101 (33%)	79 (33%)	22 (32%)
≥50 km	72 (23%)	51 (21%)	21 (31%)
Missing	9 (3%)	7 (3%)	2 (3%)
HIV WHO stage, n (%)^a^			
I & II	134 (45%)	103 (45%)	31 (46%)
III & IV	161 (55%)	124 (55%)	37 (54%)
Missing	24 (8%)	22 (9%)	2 (3%)
CD4 count cells/mm^3 a^ < 500			
	80 (33%)	52 (27%)	28 (54%)
≥ 500	164 (67%)	140 (73%)	24 (46%)
Missing	75 (24%)	57 (23%)	18 (26%)
ART regimen type, n (%) NNRTI based			50 (71%)
	205 (64%)	155 (62%)	
PI based	58 (18%)	49 (20%)	13 (19%)
DTG based	56 (18%)	45 (18%)	7 (10%)

VL, viral load, ART, antiretroviral therapy, NNRTI, non-nucleoside reverse transcriptase inhibitor, PI, protease inhibitor, DTG, dolutegravir. Results are number and column percent of those with non-missing data, missing data rows are number and column %. ^a^HIV WHO stage and CD4 measurement closest to VL testing within 6 months before and 3 months after.

### Viral suppression and associated factors

Overall, 249 (78%) adolescents had a suppressed VL. We compared demographic and clinical characteristics of ALHIV with and those without VL suppression and found them to be broadly comparable with the exception of those with suppressed VL being less likely to be female (120 (48%) vs 40 (57%)), to have disclosed their HIV status (159 (71%) vs 54 (85%)), to reside ≥50 kilometres from clinic (51 (21%) vs 21 (31%)), and more likely to have a CD4 cell count ≥500 cells/mm^3^ ((140 (73%) vs 24 (46%)) and to be on dolutegravir-based regimen (45 (18%) vs 7 (10%)) (**Table 1**).

In the modelling, the continuous variables of age and years since ART initiation were included as linear as there was no non-linearity detected. The variable presumed mode of transmission was excluded from the modelling because of a large amount of missing data (40%), and pill count and visual analog scale were not included because they were only measured in a subset of the participants. Factors associated with viral suppression were having a CD4 cell count ≥500 cells/mm^3^ (adjusted Odds Ratio (aOR) 3.48; 95% CI 1.49–8.13 versus those with a CD4 cell count <500 cells/mm^3^), and being on a dolutegravir-based regimen (aOR 13.1; 95% CI 2.50–68.7 versus those on NNRTI-based regimen). Female gender was associated with lower odds of having viral suppression (aOR 0.41; 95%CI 0.18–0.93) (**Table 2**). There was no association between viral suppression and age, years since ART initiation, education, HIV disclosure, distance from clinic or WHO stage. Self-reported good adherence with never missing a dose was associated with viral suppression (aOR 3.65; 95%CI 1.02–13.0). There was no association between viral suppression and pill box return. Results from the sensitivity analysis yielded broadly similar interpretations (**Table 2**).

**Table 2:** Association between participant characteristics and viral suppression^a^ among adolescents living with HIV.

Characteristics	Univariable		Multivariable, N = 201		Multivariable, N = 319	
	(No missing covariate values)		(No missing covariate values)		(Missing indicator used for missing covariate values)	
	OR [95% CI]^b^	Pvalue ^b^	OR [95% CI]^b^^c^	Pvalue ^b^^c^	OR [95% CI]^b^^c^	Pvalue ^b^^c^
Sex Male	Reference		Reference		Reference	
Female	0.72 [0.42, 1.23]	0.23	0.41 [0.18, 0.93]	0.03	0.59 [0.33, 1.07]	0.08
Age in years^d^	0.99 [0.92, 1.09]	0.98	0.96 [0.83, 1.12]	0.64	1.02 [0.92, 1.14]	0.71
Years since ART initiation^d^	1.01 [0.93, 1.08]	0.89	0.99 [0.87, 1.15]	0.98	1.01 [0.91, 1.12]	0.85
Education status		0.14		0.12		0.20
None	Reference		Reference		Reference	
Primary	0.84 [0.39, 1.78]		1.13 [0.34, 3.70]		1.05 [0.45, 2.46]	
Secondary	0.42 [0.16, 1.09]		0.29 [0.05, 1.58]		0.47 [0.15, 1.50]	
Disclosed HIV status		0.05		0.26		0.22
No	Reference		Reference		Reference	
Yes	0.47 [0.23, 0.98]		0.55 [0.20, 1.56]		0.51 [0.23, 1.11]	
Missing	-		-		0.73 [0.22, 2.46]	
Distance from clinic		0.19		0.13		0.41
< = 1 km	Reference		Reference		Reference	
2 - <50 km	0.85 [0.45, 1.62]		0.57 [0.21, 1.51]		0.66 [0.32, 1.34]	
≥50 km	0.54 [0.28, 1.06]		0.35 [0.13, 0.98]		0.56 [0.27, 1.18]	
Missing	-		-		1.25 [0.19, 8.22]	
HIV WHO stage		0.90		0.74		0.20
I/II	Reference		Reference		Reference	
III/IV	0.97 [0.56, 1.67]		1.16 [0.50, 2.68]		1.06 [0.57, 1.97]	
Missing	-		-		4.39 [0.88, 21.9]	
CD4 count, cells/mm^3^		0.001		0.004		0.009
< 500	Reference		Reference		Reference	
≥500	2.97 [1.58, 5.60]		3.48 [1.49, 8.13]		2.90 [1.43, 5.85]	
Missing	-		-		1.33 [0.61, 2.91]	
ART regimen type		0.16		0.003		0.09
NNRTI based	Reference		Reference		Reference	
PI based	1.25 [0.61, 2.53]		3.81 [0.86, 16.8]		1.04 [0.45, 2.38]	
DTG	2.27 [0.97, 5.34]		13.1 [2.50, 68.7]		2.96 [1.12, 7.81]	
Self-reported ART adherence, past 4 weeks		0.03		0.047		0.03
Missed ≥1 dose	Reference		Reference		Reference	
Never missed dose	2.43 [1.09, 5.43]		3.65 [1.02, 13.0]		2.59 [1.08, 6.21]	
Pill box return		0.90		0.28		0.55
No	Reference		Reference		Reference	
Yes	0.96 [0.56, 1.66]		1.60 [0.68, 3.80]		1.22 [0.64, 2.29]	

ART, antiretroviral therapy. Presumed mode of transmission (N = 191) excluded from the model because of too much missing data (missing, 40%). Pill count (n = 181) and Visual analog scale (n = 110) not considered because they are from a subset of the participants

^a^Viral suppression defined as viral load<1000 copies/ml

^b^Odds Ratios (OR), 95% confidence intervals (CI) and P-values obtained from logistic models. ^c^Adjusted for all other covariates ^d^Tested for non-linearity using polynomial models, both variables were non-significant

### Agreement between adherence measures and viral suppression

Percentage agreement between adherence assessment methods and viral suppression was 76% for self-reported adherence, 58% for pill box return, 41% for pill count and 62% for VAS with kappa values of 0.10, 0.002, -0.005 and 0.09 respectively (**Table 3**). ALHIV who reported never missing a dose on self-report adherence and those with optimal VAS adherence were more likely to be virally suppressed (230 (92%) vs 59 (84%) and 58 (66%) vs 13 (59%) respectively). Viral suppression among ALHIV who returned their pill box during clinical/drug refill visits and those with optimal pill count were not different.

**Table 3:** Agreement between adherence to ART and viral suppression among adolescents living with HIV.

Adherence to ART		Viral Load		
	Suppressed	Unsuppressed	All	Percent agreement
	(<1000 copies/ml)	(≥1000 copies/ml)	N (%)	
	N (%)	N (%)		(Kappa)
**Overall**	249 (100%)	70 (100%)	319 (100%)	
**Self-reported in the past 4 weeks**				
** Never missed a dose**	230 (92%)	59 (84%)	289 (91%)	76%
** Missed at least one dose**	19 (8%)	11 (16%)	30 (9%)	(0.10)
**Pill Box return**				
** Yes**	140 (57%)	41 (59%)	181 (57%)	58%
** No**	109 (44%)	29 (41%)	138 (43%)	(0.002)
**Pill count** ^**a**^				
** ≥90%**	95 (68%)	28 (69%)	123 (68%)	41%
** <90%**	45 (32%)	13 (31%)	58 (32%)	(-0.005)
**Visual Analog Scale**^**b**^ **Optimal (≥90%)**				
	58 (66%)	13 (59%)	71 (65%)	62%
** Sup Optimal (<90%)**	30 (34%)	9 (41%)	39 (35%)	(0.09)

ART antiretroval treatment

Results are number and column percent of those with non-missing data.

^a^among those with a pill box return, calculated as (number of pills dispensed in previous visit–number of pills returned)/number of days elapsed x100

^b^110 participants completed an in-depth adherence support questionnaire for Visual Analog Scale

## Discussion

In this study among adolescents living with HIV in rural South-western Tanzania, viral suppression rates were 78%. Strongest predictors for viral suppression were being on a dolutegravir-based regimen and having a CD4 cell count ≥500 cells/mm^3^ but also to a lesser extent self-report of never missing ART and being male. Among different adherence assessments, self-report adherence had the highest agreement with viral suppression.

The viral suppression rate of 78% among ALHIV is in line with previous data from KIULARCO, showing suppression rates of around 75% in children and adolescents enrolled in the years 2005–2016 [29]. This aligns will a study from South Africa with viral suppression rates among ALVHIV reported to be 74% in Mpumulanga district and 78% in Free State Province [6, 30]. Similarly, a multisite study among adolescents from 15 health facilities in Southern Tanzania found suppression rates in adolescents of 83% [31] and up to 88% in a national survey including participants from 36 health facilities in Tanzania in 2023 [32]. Studies from Homa Bay, Kenya reported viral suppression rates of 80% and from Kabale, Uganda of 81% [7, 8]. Compared to suppression rates in adults ranging from 81% and 89%, ALHIV are behind [1, 29]. With this, our findings further emphasize the need for action to improve services for adolescents, to ensure good individual clinical outcomes but importantly also reduce onward HIV transmission and drug resistance development. An important difference between our and other studies is that the majority of ALHIV enrolled before the rollout of dolutegravir-based regimens [31–34] which contributes to poorer suppression rates compared with dolutegravir-containing regimens [1, 31, 32, 35, 36]. Worrisome are recent reports on the emergence of viral resistance mutations against dolutegravir, which—especially in ALHIV with increased rates of poor adherence to ART—might evolve into a major problem [37–40].

The association of high CD4 cell counts with viral suppression is in line with other studies from high HIV-burdened countries [41] and with a cross-sectional study from Northern Tanzania from 2019 showing an association of higher CD4 counts with viral suppression similarly to a retrospective study from Ethiopia [41, 42], and other studies and surveys done between 2015 and 2017 in Nigeria, Eswatini, Lesotho, Malawi, Zambia and Zimbabwe [43, 44], all evidence at pointing to a good immune reconstitution for those virally suppressed.

Another factor associated with viral suppression was report of never missing an ART dose. A study from Eastern Cape province in South Africa found an association between optimal self-reported adherence and undetectable viral loads among adolescents aged 10–19 years [45]. While self-reported adherence had the best agreement (76%) with viral outcomes, the agreement was considerably lower for VAS, pill box return and pill count. This confirms data from a study conducted in Cameroon, which also found a good agreement of self-reported adherence and viral suppression of 74% [46]. However, other studies could not demonstrate a correlation between self-reported adherence and viral suppression. For example a study from an urban Tanzania reported an association of self-reported adherence with unsuppressed viral load only if combined with information on pill box return [47]. Thus, interpretation of adherence assessment remains challenging and might point towards reasons, why adolescents struggle with appropriate reporting of adherence, such as a high pressure to report good adherence, ongoing stigmatization and denial of the infection [48].

In our study, female adolescents had 59% lower odds of viral suppression compared to males. Mixed results on association between sex and viral suppression among adolescents have been reported. Studies from Cape Town, South Africa and Harare Zimbabwe found females to have lower suppression rates [13, 49], while others from Mbale, Uganda and Ehlanzeni, South Africa reported higher odds of viral suppression among female adolescents [6, 33]. The difference might arise from differences in settings, such as our cohort being located in a very rural area, where women are in more vulnerable life situations, often depending on men in terms of securing income or food, as a majority are farming.

Our study adds to the scarce literature on viral suppression among adolescents living with HIV from routine care in rural Africa. Our study has limitations. First, data on VAS was only available for a subset of participants. Second, our results may be affected by recall bias, response bias, and data quality issues, importantly missingness of data. Missing data could introduce bias, as ALHIV lost to follow-up or with unreported outcomes may have different viral suppression rates than those included, potentially leading to an overestimation of suppression rates. Third, most adolescents included in our study were not on a dolutegravir-based regimen, but on Efavirenz, which was the first-line regimen during the study. Introduction of dolutegravir-based regimens led to a better viral control in most settings [35–38]. Fourth, the viral load threshold to define viral suppression as per national guidelines during our study was 1000 copies/mL, which in the meantime in Tanzania has been lowered to 100 copies/ml, which allows to interpret results only within the context of the threshold used. Last, we could only include adolescents attending the clinic, therefore excluding those who were lost to follow-up, who would likely not be virally suppressed might have affected the suppression rate.

In conclusion, viral suppression among adolescents in this rural setting remains below the UNAIDS viral suppression goal. Being on a dolutegravir-based regimen, having a CD4 cell count ≥500 cells/mm^3^ and self-report of never missing a dose were associated with viral suppression. Currently, tools to assess adherence are unsatisfactory. Adolescence-friendly interventions such as facilitated access to clinics, addressing challenges with adherence and patient-centered care models are key to improve viral suppression. Better tools for measuring adherence among adolescents are of paramount importance in order to better predict the risk virologic failure and with this development of resistance and onwards transmission.
